# Irradiation and lithium treatment alter the global DNA methylation pattern and gene expression underlying a shift from gliogenesis towards neurogenesis in human neural progenitors

**DOI:** 10.1038/s41398-023-02560-w

**Published:** 2023-07-13

**Authors:** Christina Neofytou, Alexandra Backlund, Klas Blomgren, Ola Hermanson

**Affiliations:** 1 Department of Neuroscience, Karolinska Institutet, 171 77 Stockholm, Sweden; 2 Department of Women’s and Children’s Health, 171 77 Stockholm, Sweden; 3 Pediatric Oncology, Karolinska University Hospital, 171 64 Stockholm, Sweden

**Keywords:** Epigenetics and plasticity, Molecular neuroscience

## Abstract

Central nervous system (CNS) tumors account for almost a third of pediatric cancers and are the largest contributor to cancer-related death in children. Cranial radiation therapy (CRT) is, often in combination with chemotherapy and surgery, effective in the treatment of high-grade childhood brain cancers, but it has been associated with late complications in 50–90% of survivors, such as decline in cognition and mood, decreased social competence, and fatigue. A leading hypothesis to explain the decline in cognition, at least partially, is injury to the neural stem and progenitor cells (NSPCs), which leads to apoptosis and altered fate choice, favoring gliogenesis over neurogenesis. Hence, treatments harnessing neurogenesis are of great relevance in this context. Lithium, a well-known mood stabilizer, has neuroprotective and antitumor effects and has been found to reverse irradiation-induced damage in rodents, at least in part by regulating the expression of the glutamate decarboxylase 2 gene (*Gad2*) via promoter demethylation in rat NSPCs. Additionally, lithium was shown to rescue irradiation-induced cognitive defects in mice. Here, we show that irradiation (IR) alone or in combination with lithium chloride (LiCl) caused major changes in gene expression and global DNA methylation in iPSC-derived human NSPCs (hNSPCs) compared to untreated cells, as well as LiCl-only-treated cells. The pattern of DNA methylation changes after IR-treatment alone was stochastic and observed across many different gene groups, whereas differences in DNA methylation after LiCl-treatment of irradiated cells were more directed to specific promoters of genes, including genes associated with neurogenesis, for example *GAD2*. Interestingly, IR and IR + LiCl treatment affected the promoter methylation and expression of several genes encoding factors involved in BMP signaling, including the BMP antagonist gremlin1. We propose that lithium in addition to promoting neuronal differentiation, also represses glial differentiation in hNSPCs with DNA methylation regulation being a key mechanism of action.

## Introduction

Brain cancer is the largest contributor to pediatric cancer-related death [1]. Pharmacological, surgical, as well as radio-technical advancements in therapeutic regimens have improved prognoses over the past decades [2]. With decreasing mortality rates, however, came increases in the prevalence of long- and late-term comorbidity [3]. Survivors now face the lasting neurocognitive, psychological, and endocrine sequelae that follow intensive treatment protocols targeting the central nervous system (CNS). Neurocognitive deficits among pediatric brain tumor survivors include impaired executive functioning, cognitive plasticity, and memory [4–6]. These deficits in turn correlate to lower education, employment and quality of life (QOL) indices [4, 7]. Neurocognitive deficits occur as consequences of both intrinsic tumor effects and therapeutic action. While surgical intervention and several chemotherapeutic agents have been linked to neurocognitive injury [5, 8], cranial radiotherapy (CRT) is the regimen most associated with reduced QOL.

The mechanisms resulting in CRT-induced sequelae are multifactorial and progress synergistically over time [9]. Irradiation (IR) induces several glial, neuronal, and vascular elements that combine to harm and impede the survival of neural stem and precursor cells (NSPCs) and impair neurogenesis [10]. The areas of the brain with the highest proliferative activity are most sensitive to irradiation. The subgranular zone (SGZ) in the dentate gyrus (DG) of the hippocampus, along with the subventricular zone (SVZ) of the lateral ventricles, exhibits life-long neurogenic activity [11]. This activity is considered crucial to hippocampal functions such as memory, learning, and spatial processing [12]. Damage to SGZ NSPCs and their activity have been shown to account for much of irradiation-induced pathophysiology [13, 14]. Radiation induces neural apoptosis in addition to alterations in the hippocampal microenvironment that contribute to increased microglial inflammation, reduced NSPC proliferation, and decreased neurogenesis [9, 10, 15]. Reduced neurogenetic capacity is thought in part to be due to damage to the epigenetic methylation machinery, crucial tools for hippocampal function [16, 17]. Specifically, irradiation appears to decrease DNA-methyltransferase and histone deacetylase activity, leading to concomitant aberrations in DNA methylation patterns [18, 19].

CRT is a key component in the majority of treatment plans for pediatric brain cancers, including ependymomas; germ cell tumors; embryonal tumors, such as medulloblastoma and atypical teratoid/rhabdoid tumors; high grade gliomas (i.e., anaplastic astrocytoma, glioblastoma); and unresectable low-grade gliomas [2, 3]. Developments in radiation techniques to minimize dosage to healthy brain tissue, such as focal proton CRT, have ameliorated the degree of, but not eliminated, neurocognitive damage [20]. The refinement of radiotherapeutic technology remains a risk-reducing approach to neurocognitive sequelae, thus the need for viable treatment options is paramount.

Lithium, an alkali metal, has an established variety of applications within neuropsychiatric disease and injury. The element has demonstrated neuroprotective and regenerative effects on hippocampal NSPCs in several preclinical settings [21–23]. Studies have indicated that pretreatment with lithium before CRT attenuates radiation-induced apoptosis of hippocampal SGZ neurons and protects against associated cognitive damage in murine models [22, 24]. Though lithium appears not to protect cancer cells [25], and studies in vitro show lithium to be toxic to human pediatric brain tumor cells while preserving NSPCs [24, 26], hesitancy to unintentionally weaken the radiotherapeutic effect on tumor cells remains, in spite of findings promoting lithium as a radiosensitizer in specific medulloblastoma treatment settings [26]. Post-irradiative effects of lithium have therefore been of interest. Recently, we and our colleagues could show that post-irradiation lithium treatment in mice reversed CRT-induced NSPC damage and salvaged neurocognitive function [1].

The neuroprotective mechanisms of lithium include activation of antiapoptotic signaling pathways such as phosphatyidylinsositol 3-kinase (PI3K)/Akt, which leads to the inhibition of glycogen synthase kinase-3*β* (GSK-3*β*) [27]. When active, GSK-3*β* inhibits an array of transcriptional factors central to cell survival and proliferation [28] and impedes the WNT/*β*-catenin signaling pathway, otherwise responsible for several cell fate decisions [29]. Yet the mechanisms underlying the effects of lithium in the context of irradiation remain unclear.

In a recent study, two genes were identified as upregulated by lithium-treatment after irradiation in rodents, namely those coding for regulatory proteins Tppp, involved in microtubular stabilization and assembly, and Gad-65, involved in neuronal signaling [1]. In addition, the promoter regions of these genes were found to be hypomethylated, consistent with increased expression [1].

Epigenetic processes are central to hippocampal functionality and NSPC dynamics [16, 17]. Epigenetics involves the processes in which a select minority of genomic information is displayed and expressed while the majority remains hidden and silent [30]. Transcriptional activation and repression rely on the morphological organization of the chromatin [31]. Changes in chromatin accessibility can make a DNA region either reachable and thus transcribable (euchromatin) or unreachable, un-transcribable (heterochromatin) [30]. This chromatin re-arrangement is in part due to acetylation and deacetylation of histone groups [31–33], a process with major influence on neural differentiation [34].

Biochemical modification of DNA itself is also a major determinant, namely through methylation. The 5-positioned carbon of cytosine in the 5’-CpG-3’ dinucleotide can either be methylated (5-mC) or hydroxymethylated (5-hmC). When un- or hypomethylated, transcriptional activity is promoted. Modification and decrease in DNA methylation can be exerted by demethylases, namely the ten-eleven translocation (TET) - family [35]. The Tet enzymes mediate an oxidation pathway converting 5-mC to 5-hmC [36]. Together with methylation processes, these mechanisms constitute the epigenetic toolset necessary for innumerable developmental processes throughout life [37]. The TET-family, in particular TET3 for humans, are highly expressed within the brain and have been shown essential to neuronal development and function [35, 38]. Specifically, activity of the protein family has been deemed vital to adult hippocampal neurogenesis [35, 38]. Furthermore, TET-coupled demethylation has been demonstrated to be involved in neuroprotective processes following neuronal damage [39, 40] as well as WNT signaling activity and thus various aspects of differentiation in neural progenitor cells [41].

In this study, we employed gene expression and DNA methylation assays to study the effects of lithium in irradiated human neural stem and progenitor cells (hNSPCs). A single dose of discontinued lithium treatment after irradiation in hNSPCs showed promise in promoting neuronal differentiation while simultaneously inhibiting gliogenesis, with regulation of DNA methylation being a key mechanism of action.

## Materials and methods

### Reprogramming and maintenance of human neural stem and progenitor cells (hNSPCs)

Human induced pluripotent stem cell (iPSC)-derived hNSPCs were provided by the iPS Core Facility at Karolinska Institutet. Dermal fibroblasts procured from skin biopsies were reprogrammed into iPSCs using virus - free synthetic mRNA (Stemgent, StemRNA 3rd Generation Reprogramming Kit Cat# 00–0076) and pushed to hNSPC fate development following the Dual-SMAD inhibition protocol [42]. Neural rosettes were extracted and cultured in medium enriched with epidermal growth factor (EGF) and fibroblast growth factor-2 (FGF2). hNSPC cultures were grown in T12.5, T25 and T75 sterile flasks, coated with 10% Poly-L-Ornithine (Sigma-Aldrich, Cat# P4957) and 0.2% Laminin (Sigma-Aldrich, Cat# L2020), at 37 °C in a 5% CO2-humidified incubator. Cells were cultured in Dulbecco’s Modified Eagle Medium: Nutrient mixture F-12 (DMEM/F12), GlutaMAX (Thermo Fisher Scientific, Cat# 10565018) supplemented with 1% Penicillin – Streptomycin (Pen/Strep) (10,000 U, Life technologies, Cat# 15140-122), 1% N2 (100x, Life Technologies, Cat# 17502001), 0.1% B27 (50x, Life Technologies, Cat# 12587-010), 0.1% EGF (10 ng/μl, R&D Systems, Cat# 236-EG), and 0.1% FGF2 (10 ng/μl, R&D Systems, Cat# 233-FB). Growth factor supplementation maintained proliferative activity without differentiation. Half of the medium volume was changed daily, and cultures were split at 60–80% confluence every 3-4 days.

### Irradiation and lithium treatment

Cells were seeded at 250,000 cells/well on pre-coated 12-well cell culture plates and expanded in medium overnight to ~60% confluence. Subsequently, cells were irradiated in an CIX2 Xstrahl X-Ray Irradiator with Aluminum filter at a focus-to-skin distance (FSD) of 40 cm and with an energy of 195 kV, 10 mA for 2 min and 58 s, for a received dose of 4 Gray (4 GY), as described before in mouse [22] and rat [1] NSPCs. The presence of DNA damage after 4 GY irradiation was confirmed on hNSPC cultures with γH2AX staining (data not shown). To allow for assessment of irradiation damage, cells remained in the incubator for 1 h. Pre-warmed DMEM/F-12 was mixed with the appropriate amount of LiCl powder (Sigma-Aldrich, Cat# L9650-100G), passed through a 0.22 μm filter, and supplemented with 1% Penicillin-Streptomycin, 1% N2, 0.1% B27, 0.1% EGF, and 0.1% FGF2 to produce 3 mM Lithium Chloride (LiCl) hNSPC medium, as previously described in mouse [22] and rat [1] NSPCs.

Control hNSPC-medium was selectively replaced with LiCl, to achieve the desired experimental conditions. Cells of applicable conditions were treated with 3 mM LiCl for 3 h, washed once with warm medium, and allowed to expand for 48 h in standard medium at 37 °C in a 5% CO_2_-humidified incubator.

### RNA isolation and gene expression analysis

RNA isolation was performed using the RNeasy Mini Kit (Qiagen, Cat# 74104), per manufacturer instructions. Reverse transcription (RT)-PCR was completed on the isolated RNA using the High-Capacity cDNA Reverse Transcription Kit (Life Technologies, Cat# 4368814) according to the manufacturer’s protocol. qPCR on the obtained cDNA was performed with Platinum SYBR Green qPCR SuperMix-UDG Kit (Life Technologies, Cat# 11733038) in an Applied Biosystems 7300 Real-Time qPCR System. Transcripts were quantified using either Standard curve-based or Delta-Delta-CT-based qPCR. Housekeeping gene (GADPH) was used to normalize expression levels. For the gene expression analysis, two-way ANOVA was used, followed by a paired Student’s t-test if appropriate; the analysis was done in GraphPad Prism (La Jolla, CA, USA). A significance level was set at *p* < 0.05. For differential gene expression analysis, the Affymetrix Whole Transcript Assay with the Clariom S cartridge for human samples (*n* = 2, BEA core facility, Sweden) was used.

### DNA isolation and methylation analysis

DNA isolation was performed following the DNeasy Blood and Tissue Kit (Qiagen, Cat# 69504). DNA samples were sent to Bioinformatics and Expression Analysis (BEA) Core Facility, Karolinska Institute, for methylation array analysis. Single CpG sites were examined for changes in methylation status using Illumina Infinium MethylationEPIC Bead Chip (*n* = 2). Methylation values measured in beta (b, b = 1: 100% CpG methylation, b = 0: 0% CpG methylation) were compared between conditions to generate a DeltaBeta value. Gene Ontology analysis was completed on genes located in the promoter sequence and with DeltaBeta values ≤ −0.05. GO term analyses were done using the PANTHER 17.0 Classification System [43] (Los Angeles, CA, USA) operating on the GO knowledgebase and with WebGestalt [44], with the following parameters of analysis: enrichment method Over-Representation Analysis (ORA), enrichment category gene ontology, filters biological process and molecular function, Fisher’s exact test, false discovery rate (FDR) < 0.05.

## Results

### IR and lithium alter the gene expression profile of hNSPCs

**Fig. 1:**
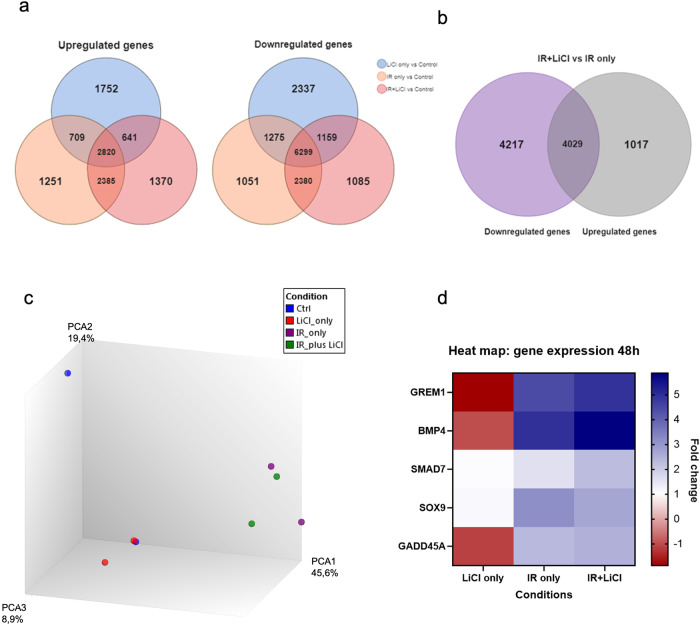
Irradiation and lithium treatment alters the gene expression profile of hNSPCs. When comparing two groups, a gene with a fold change above 1.1 was considered upregulated, below 0.9 – downregulated, *p* < 0.05. Genes with a fold change of less than or equal to 1.1 and higher or equal to 0.9 were deemed unchanged. To obtain the number of upregulated or downregulated genes the (**a**) fold change was obtained by normalizing each condition to control, and the (**b**) fold change was obtained by comparing IR + LiCl and IR only and normalizing the results to control samples. **c** Principal component analysis plot of the gene expression data in hNSPCs for the treatment groups Control, LiCl only, IR only and IR + LiCl. **d** Expression profile of selected genes presented in a Heat map.

To investigate the effect of irradiation and lithium on gene expression in hNSPCs, RNA samples were subjected to differential gene expression analysis. A total of 21,453 genes were investigated in the analysis. To determine the number of differentially expressed genes, fold changes obtained by comparing two experimental groups at a time with each other, namely Control, LiCl only, irradiation (IR) only and irradiation and lithium chloride (IR + LiCl), were used as indicators and the results are summarized in the form of Venn diagrams in Fig. 1a, b. By comparing both Venn diagrams presented in Fig. 1a, it was noted that the LiCl group had more differentially downregulated genes compared to the control, while more genes appear to be upregulated in the IR only and IR + LiCl groups, compared to the control. Comparison of the differentially expressed genes in the groups IR only and IR + LiCl revealed that there were 4029 genes that show differential expression pattens compared to the control, but a shared expression pattern between IR only and IR + LiCl, whereas there are ~1017 upregulated and 4217 downregulated genes in the IR + LiCl group compared to the IR only group (Fig. 1b).

Principal component analysis (PCA) of the gene expression data showed the formation of two distinct clusters suggesting major differences of gene expression between irradiated and non-irradiated groups (Fig. 1c). It can be deduced from the PCA plot that the largest contributor to gene expression changes was irradiation itself and that LiCl alone did not lead to significant gene expression changes in hNSPCs (Fig. 1c). Further analysis of the gene expression data pointed to several genes being significantly differentially expressed in the IR only and IR + LiCl group, with 1017 genes being upregulated in the IR + LiCl group compared to IR only and 4217 downregulated genes, some of which are presented in a heatmap in Fig. 1d.

It has already been shown that lithium indirectly inhibits GSK-3*β* and can thus lead to the activation of the WNT/*β*-catenin signaling pathway, which plays a crucial role in neurogenesis [27–29]. We investigated the gene expression levels of several factors involved in WNT signaling, e.g., WNT3a, GSK3 *β*, *β*-catenin but found no significant differences between groups that had been exposed to LiCl or not after irradiation (data not shown). Our data point to potentially different modes of action for lithium after irradiation.

### IR and lithium alter the DNA methylation profile of hNSPCs in gene promoter regions

**Fig. 2:**
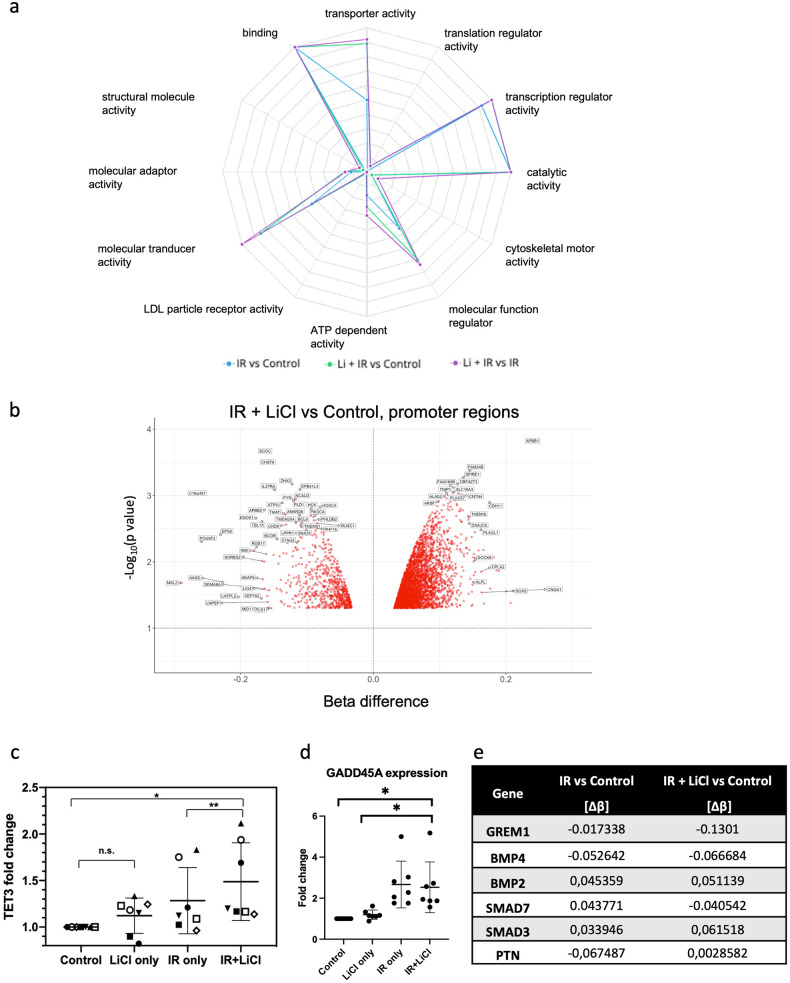
Effects of irradiation and lithium treatment on the DNA methylation status of hNSPCs. **a** Radar graph depicting proportionate distributions of hypomethylated gene promoters (5’ UTR) across first-tier GO categories per treatment comparison. **b** Volcano plot indicating genes with the most significant change in methylation between IR + LiCl and Control, for the promoter regions (5’ UTR). **c**–**d** Gene expression fold change compared to untreated and normalized to *GAPDH*, for genes involved with control of methylation, namely *TET3* (*n* = 7) (**c**) and *GADD45A* (*n* = 7) (**d**). One-way ANOVA and paired *t*-test, *p < 0.05, **p < 0.005. Error bars represent mean and SD. **e** Table of beta value difference of the promoter regions (5’ UTR) of selected genes between the IR only group and the IR + LiCl group compared to Control.

The effects of irradiation and lithium on the DNA methylation status of hNSPCs were studied with the use of Illumina Infinium MethylationEPIC Bead Chip and the results were analyzed for the gene promoter regions, as depicted in Fig. 2. In Fig. 2a, a radar graph depicts the proportionate distributions of hypomethylated gene promoters across first-tier gene ontology (GO) categories per treatment comparison. Irradiation alone was a strong inducer of DNA methylation alterations across the genome and across many GO terms, but the combinatorial treatment of IR + LiCl had a stronger correlation compared to IR only to the GO terms “transcription regulation activity”, “transporter activity”, “DNA binding”, “molecular function regulator” and “molecular transducer activity” (Fig. 2a).

The volcano plot (Fig. 2b) reveals the genes with the most significant change in methylation of promoter regions between IR + LiCl and Control. Most differentially hypo-methylated gene promoters are to the left while most differentially hyper-methylated genes are to the right. Genes with the most significant change in methylation are toward the top. Some of the top hits include genes known to regulate proliferation and differentiation of neural progenitor cells, for example the transcription factor POU Class 6 Homeobox 2 (*POU6F2*), which was hypomethylated in the IR + LiCl compared to Control. On the contrary, the genes with hypermethylated promoter regions, which are plotted to the right of the volcano plot (Fig. 2b) include genes encoding for transcription factors involved in the control of gliogenesis, for example *SOX5*, the downregulation of which is known to be necessary for the progression of neuronal differentiation. A similar trend, though less clear, is followed when the DNA methylation patterns of the entire genome is taken into account, depicted for all group comparisons in the Supplementary Fig. S1. For example, the top hits when comparing the global methylation patterns of the IR + LiCl group compared to control include *POU6F2* and the nucleoporin 35 (*NUP35*), which both were hypomethylated after irradiation and lithium treatment compared to control, and axon regeneration-associated genes, such as the small protein-rich protein 1A (*SPRR1A*), which was hypermethylated (Supplementary Fig. S1e).

The expression of two genes involved in the control of methylation, *TET3,* and *GADD45A*, was found to be upregulated in hNSPCs by IR and LiCl treatment compared to control (Fig. 2c, d). As a member of the ten-eleven translocation (TET) gene family, TET3 is a dioxygenase that catalyzes the conversion of 5-methylcytosine (5mC) into 5-hydroxymethylcytosine (5hmC), which thus could be an important factor regarding the effects of LiCl in irradiated hNSPCs. In addition, GADD45A (Growth Arrest and DNA Damage inducible Alpha) has also been associated with DNA demethylation and has been shown to interact with members of the TET-family [45]. A role for TET3 and/or GADD45A could be to control the DNA methylation pattern changes in irradiated and lithium treated hNSPCs compared to Control, shown for selected gene promoters involved in the control of gliogenesis (namely *GREM1, BMP4, SMAD7, PTN*) in the table in Fig. 2e.

### IR and lithium lead to promoter region demethylation in genes linked with neurogenesis and to *GAD2* upregulation

**Fig. 3:**
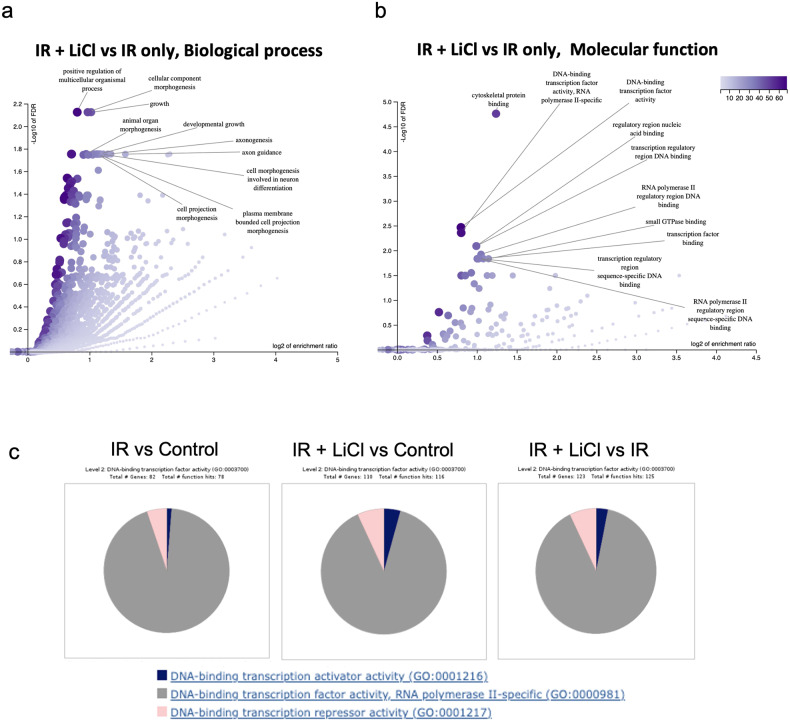
Irradiation and lithium treatment alter the DNA methylation status of gene promoters important for neurogenesis and control of transcription in hNSPCs. **a**–**b** Volcano plot depicting the top enriched gene ontology terms in the category (**a**) “Biological process” and (**b**) “Molecular function”, for the genes with significantly hypomethylated promoter regions in the IR + LiCl group vs the IR only group (*n* = 510 genes, with deltaBeta <−0.05, *p* < 0.05 in 5’ UTR region), made with WebGestalt (parameters of analysis: enrichment method: ORA, enrichment category: geneontology, Fisher’s exact test, FDR < 0.05). **c** Pie charts depicting GO term analysis on the DNA binding transcription activity category. Presented in order, 82 demethylated genes (IR vs Control), 110 (LiCl + IR vs Control), 123 (LiCl + IR vs IR).

The importance of the control of demethylation on gene promoters involved in neurogenesis and axonogenesis in irradiated and LiCl treated hNSPCs is also reflected in Fig. 3. Gene ontology term analysis of the genes with significantly hypomethylated promoter regions in the IR + LiCl group, compared to the IR only group (*n* = 510 genes, with deltaBeta <−0.05, *p* < 0.05 in 5'UTR region), revealed that genes involved in axon guidance, axonogenesis and cell morphogenesis involved in neuron differentiation, were poised for transcription in the IR + LiCl group, when filtered by the term “Biological process” (Fig. 3a, Supplementary Fig. 2b). When the same analysis was performed, this time with the term “Molecular function”, it was shown that gene promoters involved in transcription regulation were demethylated in the IR + LiCl group, compared to the IR only group (Fig. 3b, Supplementary Fig. 2c).

Both irradiation alone and lithium combined with irradiation led to global changes in DNA methylation status when compared to control. As observed in the PCA plot of the normalized beta values of each sample in Supplementary Fig. S2a, the Control and LiCl only treated groups of each independent experiment cluster close together and far away from their corresponding IR only and IR + LiCl counterparts. While irradiation alone is a great inducer of DNA methylation changes in hNSPCs, the quantity of genes with notable demethylation was greater when comparing lithium treatment following irradiation with irradiation only (Fig. 3c). Lithium treatment of non-irradiated, uninjured cells has been shown to have a considerably attenuated effect in contrast to treatment of irradiated cells [1, 22]. This tendency is upheld in our data; we found 662 genes with hypomethylated promoter regions and DeltaBeta values < −0.05 when comparing LiCl with Control whereas 1626 genes in similar localization were demethylated when comparing LiCl following irradiation with control, and 1708 when comparing LiCl following irradiation with irradiation alone (data not shown). By focusing the analysis on gene promoters which are found to be hypomethylated in pairwise comparisons under the GO term “DNA binding transcription activity”, we found 82 demethylated gene promoters in IR vs Control, 110 demethylated gene promoters in IR + LiCl vs Control and 123 demethylated gene promoters in IR + LiCl vs IR (Fig. 3c).

These, taken together, suggest that after the major re-organization of DNA methylation by irradiation alone, the addition of lithium further influences the pattern of DNA demethylation on specific promoters, including promoters of genes involved in transcription. On the contrary, the pattern of DNA methylation changes induced by IR alone is less specified to defined functions, but rather stochastic across many GO term categories (Figs. 2a and 3c).

**Fig. 4:**
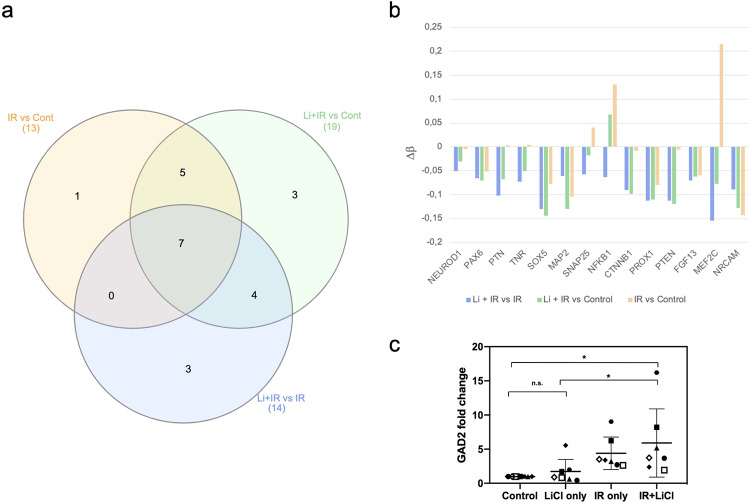
Specific effects of irradiation and lithium treatment on hNSPCs, for genes linked to neurogenesis. **a** Venn diagram of distribution of neurogenesis-linked demethylated gene promoters (5’ UTR) among treatment comparisons. **b** Bar graph depicting delta beta values for 14 neurogenesis-associated gene promoters (5’ UTR) corresponding to Li + IR vs IR. **c** Gene expression fold change compared to untreated and normalized to *GAPDH*, for *GAD2* (*n* = 7). One-way ANOVA and paired *t*-test, *p < 0.05, **p < 0.005. Error bars represent mean and SD.

Since one of the top terms for processes in which genes with demethylated promoter regions in the IR + LiCl group compared to IR only was “cell morphogenesis involved in neuron differentiation”, a more thorough analysis for hypomethylated promoters of neurogenesis-linked genes was performed and is displayed in Fig. 4. Fourteen genes associated with neuronal differentiation and characteristics (*NEUROD1, PAX6, PTN, TNR, SOX5, MAP2, SNAP25, NFKB1, CTNNB1, PROX1, PTEN, FGF13, MEF2C, NRCAM*) were found to be demethylated between IR + LiCl treatment and IR only (Fig. 4a, b, Table S3), while LiCl treatment alone produced lower or no demethylation for these genes compared to control (Fig. 4b). The neurogenesis-linked genes with DeltaBeta < −0.05 were cross-referenced with pre-existing microarray data from transcriptome analysis (BEA Core Facility, Sweden). Of the 14 genes, 4 (namely *MAP2, NRCAM, PTEN* and *PROX1*) were also found to be upregulated in the differential gene expression dataset between IR + LiCl treatment and IR only (data not shown). *MAP2*, encoding for the microtubule-associated protein 2, was the only neurogenic marker that demonstrated hypomethylated status after lithium treatment alone, when compared to IR only (data not shown). Its methylation status was also impacted dramatically by irradiation only, and even more altered by the addition of lithium (Table S3). Due to their variability, these changes are likely to be unspecific.

Most importantly, *GAD2*, the human homolog of the murine *Gad2* gene, was found to be significantly upregulated upon irradiation and lithium treatment compared to Control and LiCl only (Fig. 4c), suggesting a similar mechanism of action in human cells as the one previously observed in rodents [1].

### IR and lithium lead to promoter region demethylation in genes linked with negative regulation of gliogenesis

**Fig. 5:**
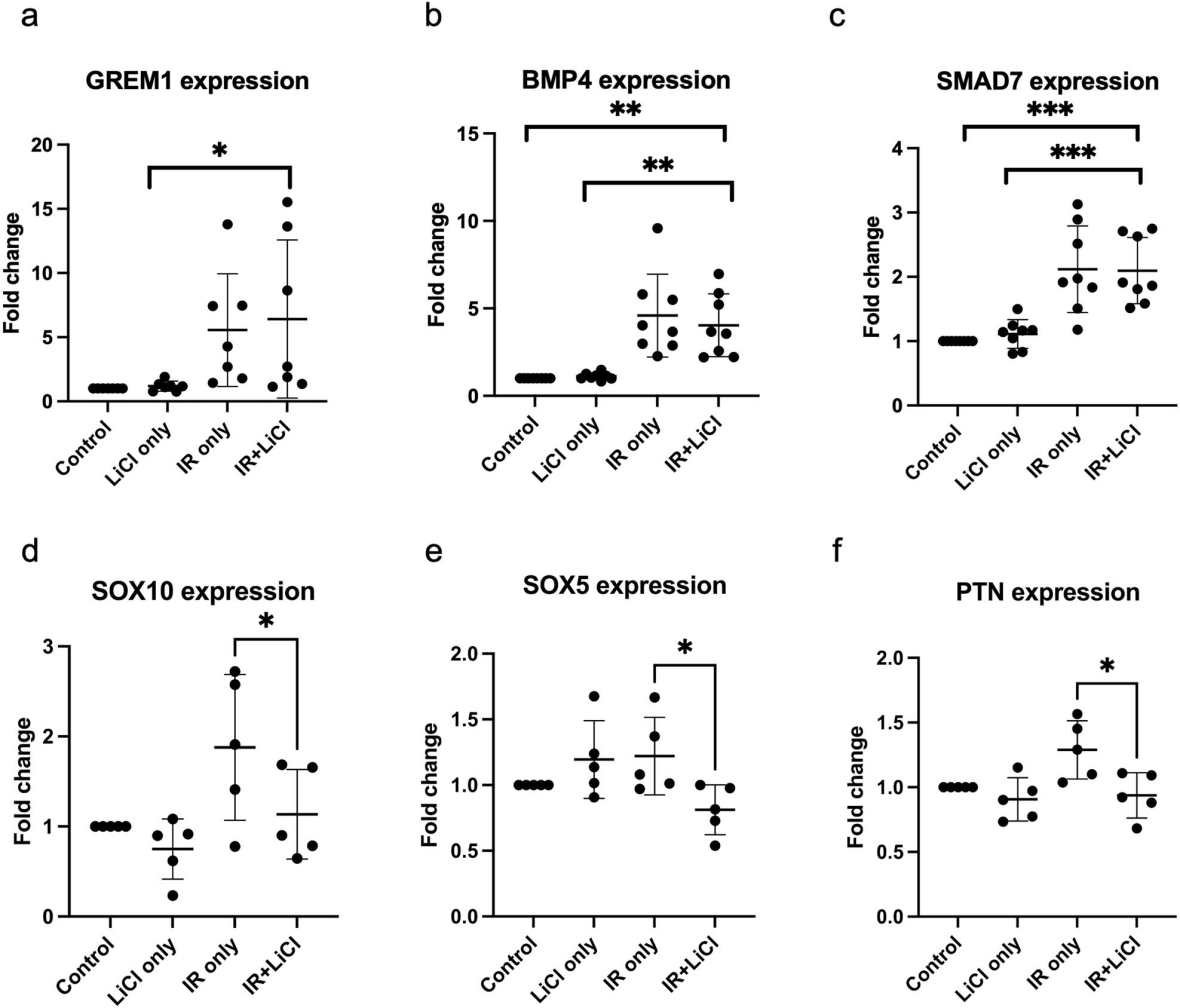
Specific effects of irradiation and lithium treatment on hNSPCs, on gene expression, for genes linked to negative regulation of gliogenesis. Gene expression fold change compared to untreated and normalized to GAPDH, for selected genes, **a** *GREM1* (*n* = 7), **b** *BMP4* (*n* = 8), **c** *SMAD7* (*n* = 8), **d** *SOX10* (*n* = 5), **e** *SOX5* (*n* = 5), **f** *PTN* (*n* = 5). One-way ANOVA and paired *t*-test, *p < 0.05, **p < 0.005. Error bars represent mean and SD.

Irradiation and lithium treatment of hNSPCs also leads to upregulation of the gene expression of *GREM1* compared to Control and LiCl only. *GREM1* encodes for gremlin1, an extracellular inhibitor of BMP signaling (Fig. 5a). Subsequently, the gene expression patterns of BMP-pathway associated genes which appeared to be differentially expressed in the microarray were investigated, including *BMP4* (Fig. 5b) and *SMAD7* (Fig. 5c), which were found to be upregulated compared to Control and LiCl only. A trend for downregulation of these genes in the IR + LiCl group was observed compared to the IR only group but was not statistically significant. The DNA methylation patterns of these genes in the group IR + LiCl are shown in relation to the beta value in the IR only and the Control group in Fig. 2e and Table S3.

The promoter regions of several more genes involved in gliogenesis, namely *DAB1, DICER1, DUSP10, LINGO1, PTN, SMAD3, SOX10, SOX5, ATF3, ATXN1, BMP2, CNTFR, DAG1,* and others were found to be hypomethylated in irradiated hNSPCs and less hypomethylated in irradiated and LiCl treated cells, as seen by analysis of the methylation array results (Table S3). Out of these, *SOX10*, *SOX5,* and *PTN* were also found to be upregulated by IR only and expressed to levels similar to the control in irradiated and LiCl treated hNSPCs (Fig. 5d, e, f respectively).

## Discussion

Significant strides have been made towards making pediatric brain cancer a curable diagnosis, yet increased rates of survival have showcased the long- and late-term neurocognitive sequelae of the available treatment modalities, in particular cranial radiotherapy. Damage to NSPCs in the DG of the hippocampal SGZ, a unique focus of lifelong neurogenesis, are presumed to play a leading role in the pathophysiology of post-irradiative neurocognitive deficits. Clinical approaches for addressing the neurocognitive deficits in pediatric brain cancer survivors are varied; the development of novel radiotherapeutic techniques with precision-based algorithms offer hope of ameliorated side effects [20], while promising preclinical studies lay the groundwork for recent and ongoing clinical trials employing, among others, metformin [46], memantine [47], and physical exercise [48] as regenerators of hippocampal neurogenesis, without a clear solution so far. Studies both in vitro and in murine models have demonstrated the ability of lithium to selectively rescue NSPCs and their functionality after irradiation [1, 21, 22, 24, 25, 49].

Here we demonstrated that irradiation and lithium treatment of hNSPCs contributes to a distinct transcriptomic profile, especially compared to control and LiCl only (Fig. 1). The irradiation treatment alone is the biggest inducer of transcriptomic alterations, consistent with other studies [40, 50], and the addition of lithium after irradiation treatment has a significant, yet less robust effect on gene expression. Notably, irradiation and lithium treatment of hNSPCs lead to an upregulation of *GAD2* (Fig. 4c), which encodes a rate-limiting enzyme (GAD2) involved in GABA inhibitory neurotransmitter synthesis [51]. In previous studies, lithium increased *Gad2* expression after irradiation of rodent neural progenitors [1]. Moreover, increased *GAD2* expression after lithium treatment was also followed by the decrease in methylation (Table S3), implying that the gene expression was affected by epigenetic changes. GABA-mediated signaling has been shown to play an essential role in regulating proliferation, differentiation, and survival of neural progenitors monitoring normal brain development. On the contrary, improper GABA synthesis has been shown to cause many neurological diseases [51]. Other neurogenesis-associated genes were also found to be upregulated (*MAP2, NRCAM, PTEN*, and *PROX1)*, with the promoter of *MAP2* also being hypomethylated in IR + LiCl treated cells compared to the control. One study on ischemic stroke in rodents, demonstrated an upregulation of *MAP2* in the penumbral—injured but not necrotic—region, which was attributed to ongoing neuronal repair [52]. Our findings suggest that lithium can potentially restore normal brain development after irradiation by increasing GABAergic signaling and thereby enhancing neuronal differentiation and survival.

**Fig. 6:**
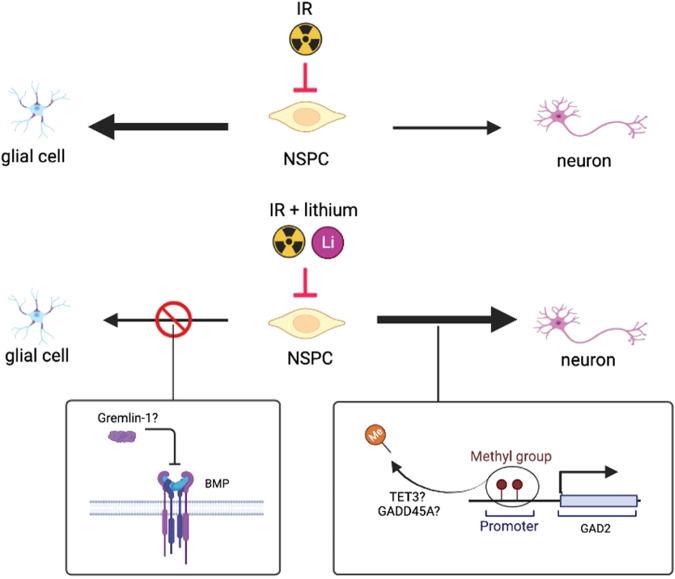
Proposed mechanism of action of lithium treatment in irradiated hNSPCs. A key pathology of the irradiated hippocampus is a skewed developmental process that favors gliogenesis over neurogenesis. Post-irradiative lithium treatment led to increased demethylation of several genes related to a functional neurogenesis and negative control of gliogenesis. The promoter demethylation was also accompanied by transcriptional upregulation of developmentally important genes, refocusing this developmental equilibrium towards neurogenesis, such as *GAD2*, and away from gliogenesis, such as *GREM1*, encoding gremlin1, a BMP inhibitor. The selective mechanism of methylation alterations after IR + LiCl treatment could be controlled by *TET3* and *GADD45A*, which were also found to be upregulated after irradiation and lithium treatment in neural progenitor cells compared to the control. Created with BioRender.com.

Interestingly, we revealed that irradiation and lithium treatment is involved in negative regulation of gliogenesis in hNSPCs, as demonstrated by the upregulation of *GREM1*, encoding a known BMP inhibitor, gremlin1, in IR + LiCl-treated cells (Fig. 5a). The BMP pathway plays an essential role in stem cell fate; in the neurogenic regions of the brain, BMP pathway can promote glial fate commitment [53]. BMP4 is a positive regulator, while SMAD7 (intracellular) and gremlin1 (extracellular) are negative regulators of the BMP pathway [53]. Inhibition of the BMP pathway through gremlin1 binding to BMPs has been shown to promote neuronal specification [54, 55]. In this study on human neural progenitors, we observed increased *GREM1* expression in the irradiated and LiCl-treated group (Fig. 5a). A fivefold increase in *GREM1* levels suggests that one mode of how lithium can exert its effects is by inhibiting the BMP pathway and thereby decreasing astroglial differentiation. This result is also strengthened by the decrease in expression of other gliogenesis marker genes, such as *SOX10, SOX5,* and *PTN* after irradiation and lithium treatment (Fig. 5d–f), in parallel to the appropriate DNA methylation pattern changes (Table S3). The proposed mechanism of action of lithium in irradiated hNSPCs is summarized in Fig. 6.

In summary, we present the global and targeted effects of lithium and irradiation on gene expression and on DNA methylation following irradiation of hNSPCs. We demonstrate that lithium treatment following irradiation results in the demethylation, poising, and thereof increased transcription susceptibility and potential upregulation, of select genes central to neurogenesis and NSPC survival. In addition, our results point to potential mechanisms with which lithium could enact these effects on DNA methylation status, by implicating TET3 and GADD45A.

## Conclusions

This study has demonstrated that irradiation alters the DNA methylation profile of hNSPCs, poising gene promoter regions important for transcription factor binding, an effect that is further specified after lithium treatment. Several studies have established that lithium promotes NSPC survival and proliferation post-irradiation [1, 21, 22, 24, 49]; our data suggest that the effect of lithium in irradiated NSPCs is dependent on epigenetic regulation.

A key pathology of the irradiated hippocampus is a skewed developmental process that favors gliogenesis over neurogenesis [1]. Post-irradiative lithium treatment led to increased demethylation of several genes related to a functional neurogenesis and negative control of gliogenesis. The promoter demethylation was also accompanied by transcriptional upregulation of developmentally important genes, refocusing this developmental equilibrium towards neurogenesis, such as *GAD2*, and away from gliogenesis, such as *GREM1*, *SOX10, SOX5* and *PTN* can thereof be central to the positive effects of lithium. The promoters of these genes were found to be differentially methylated in irradiated and lithium treated cells, with patterns consistent with their expression profiles compared to the other experimental groups. This selective mechanism of methylation alterations after IR + LiCl treatment could be controlled by *TET3* and *GADD45A*, which were also found to be upregulated after irradiation and lithium treatment in neural progenitor cells compared to the control.

Lithium is presumed to have a range of still poorly understood mechanisms through which it restores damage of the irradiated brain [1, 24]. Shining a light on the epigenetic role of lithium in favoring neurogenesis over gliogenesis in irradiated human neural progenitors further strengthens the arguments for its clinical use.

## Supplementary information

Supplemental Material Legends

Supplementary Figure S1

Supplementary Figure S2

Supplementary Table S3

## Data Availability

The array datasets are deposited in the Gene Expression Omnibus (GEO) database (https://www.ncbi.nlm.nih.gov/geo/), under the accession numbers GSE234353 and GSE236202, for the gene expression array and the DNA methylation array, respectively.
