# Supplementary material for: Irradiation and lithium treatment alter the global DNA methylation pattern and gene expression underlying a shift from gliogenesis towards neurogenesis in human neural progenitors
Source: Transl Psychiatry. 2023 Jul 13;13:258. doi: 10.1038/s41398-023-02560-w (PMC10345108; doi:10.1038/s41398-023-02560-w)
Supplement: Supplementary file 1 — Supplemental Material Legends [file 41398_2023_2560_MOESM1_ESM.docx]

# Supplementary Material

## Supplementary figure legends

***Supplementary Figure S1. Irradiation alters the global DNA methylation profile of hNSPCs.*** *Volcano plots indicating genes with the most significant change in methylation between a) the IR only and Control, b) LiCl only and Control, c) IR only and LiCl only, d) IR + LiCl and IR only, e) IR + LiCl and Control, and f) IR + LiCl and LiCl only.*

***Supplementary Figure S2. Comparison of the DNA methylation status of hNSPCs in the IR + LiCl group to IR only reveals distinct biological and molecular functions.*** *a) Principal component analysis plot of the normalized beta values of the treatment groups Control, LiCl only, IR only and IR + LiCl of hNSPCs. b, c) Bar plots depicting the top enriched gene ontology terms in the category b) “Biological process” and c) “Molecular function”, for the genes with significantly hypomethylated promoter regions in the IR + LiCl group vs the IR only group (n= 510 genes, with deltaBeta < -0.05, p < 0.05 in 5’ UTR region), made with WebGestalt (parameters of analysis: enrichment method: ORA, enrichment category: geneontology, Fisher’s exact test, FDR < 0.05).*

***Supplementary Table S3. Table of beta value difference of the promoter regions (5’ UTR) of selected genes between the IR only group, and the IR + LiCl group, compared to Control.***
