# Supplementary figures and images for: Irradiation and lithium treatment alter the global DNA methylation pattern and gene expression underlying a shift from gliogenesis towards neurogenesis in human neural progenitors
Source: Transl Psychiatry. 2023 Jul 13;13:258. doi: 10.1038/s41398-023-02560-w (PMC10345108; doi:10.1038/s41398-023-02560-w)

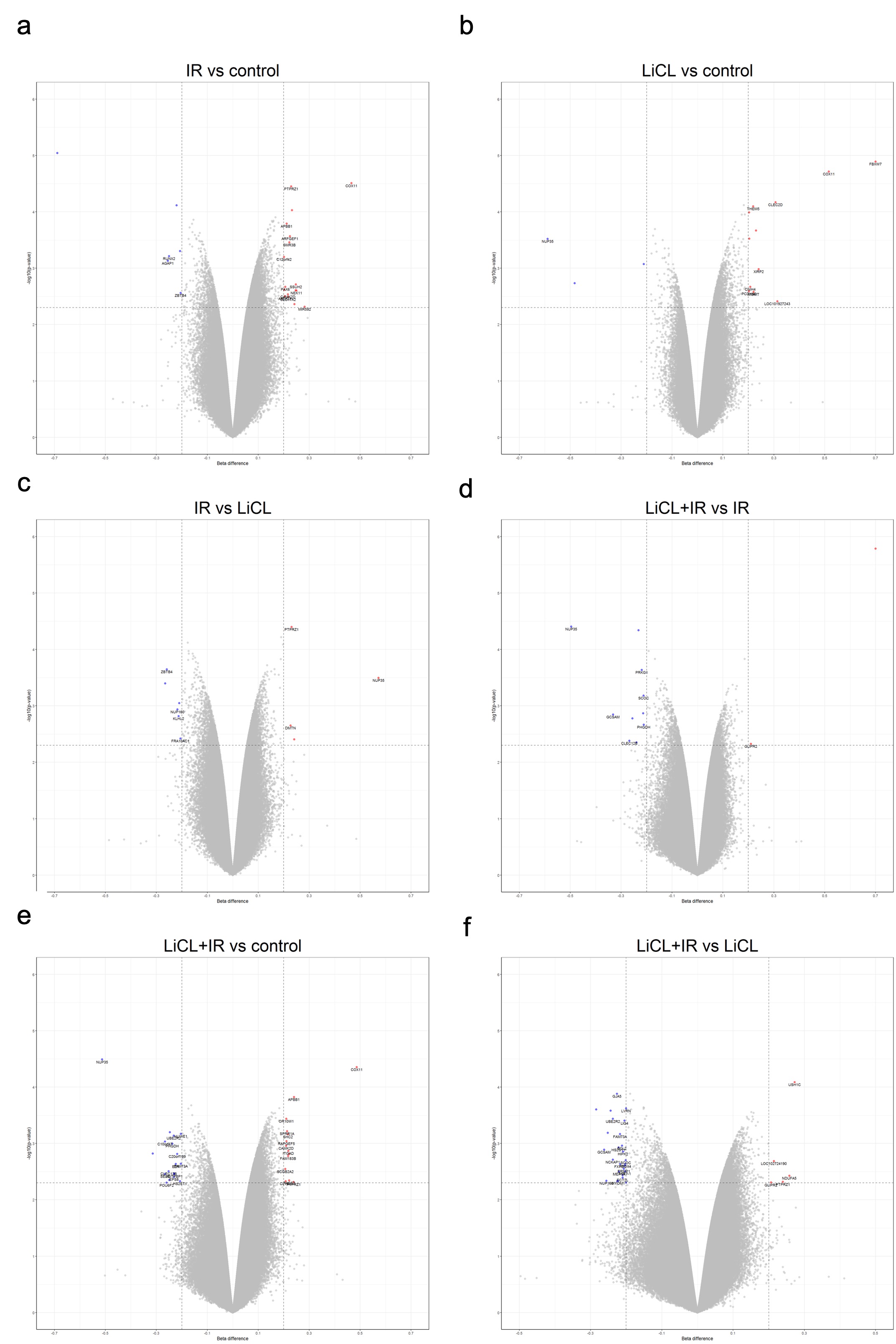

Supplement: Supplementary file 2 — Supplementary Figure S1 [file 41398_2023_2560_MOESM2_ESM.jpg]

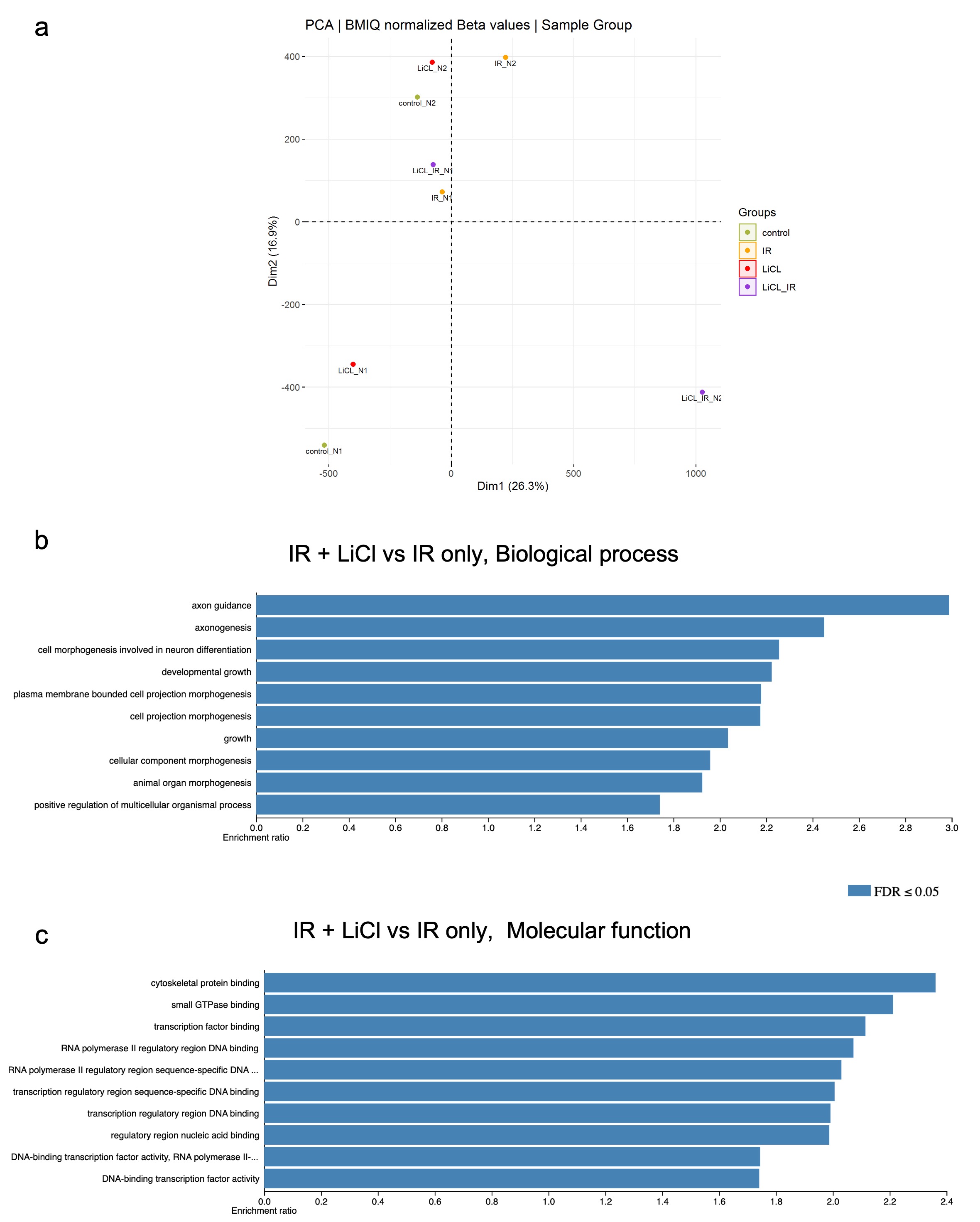

Supplement: Supplementary file 3 — Supplementary Figure S2 [file 41398_2023_2560_MOESM3_ESM.jpg]

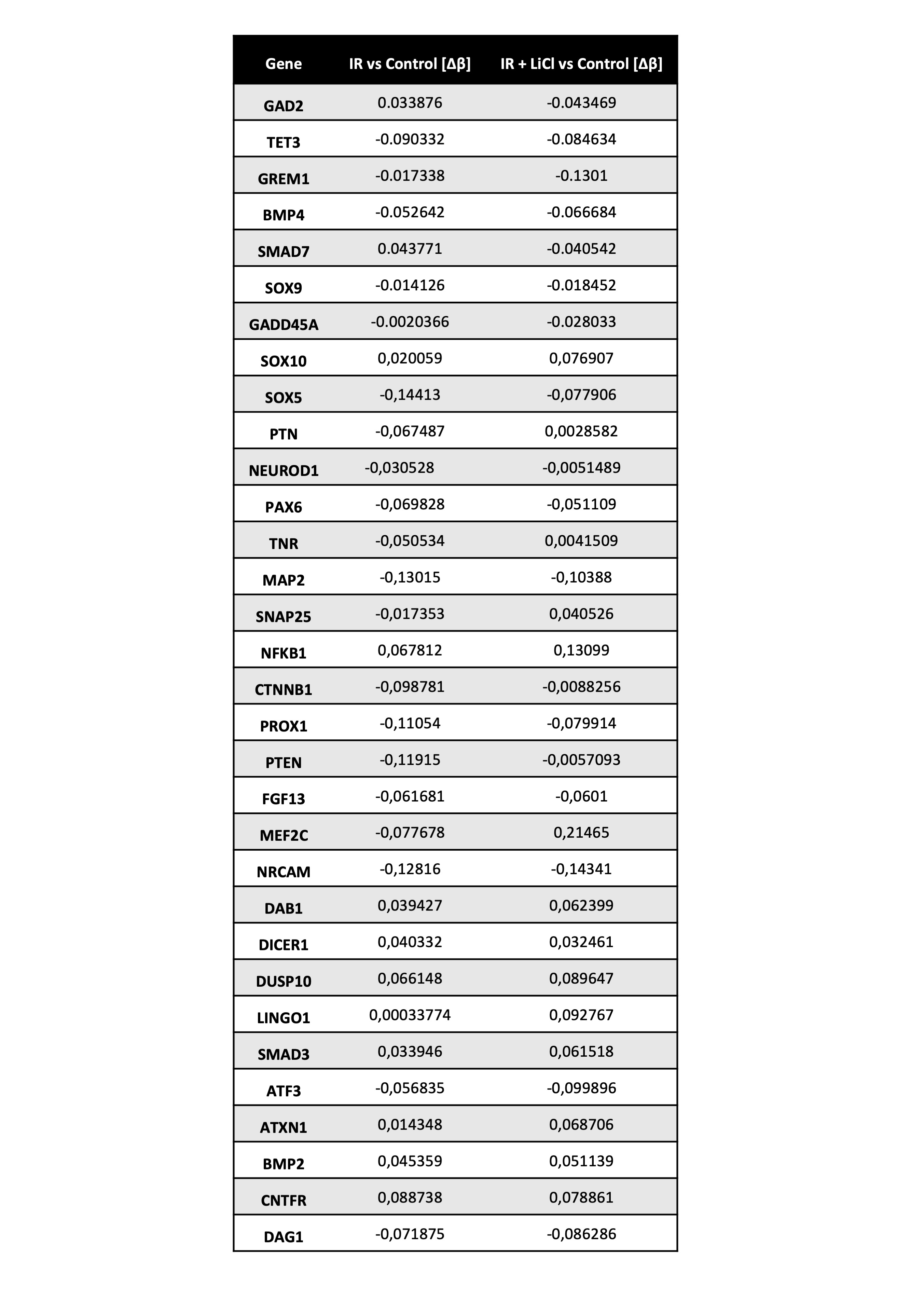

Supplement: Supplementary file 4 — Supplementary Table S3 [file 41398_2023_2560_MOESM4_ESM.jpg]
